# Münsteraner Fahrradunfälle im Wandel der Zeit: Analyse der Entwicklung der Fahrradunfälle in den letzten 10 Jahren

**DOI:** 10.1007/s00113-022-01287-5

**Published:** 2023-01-30

**Authors:** Désirée Schlautmann, Michael Raschke, Udo Weiss, Britta Wieskötter, Johanna Ueberberg, Christian Juhra

**Affiliations:** 1 St. Franziskus Hospital Münster, Hohenzollernring 70, 48145 Münster, Deutschland; 2 Universitätsklinikum Münster, Münster, Deutschland; 3 Polizeipräsidium Münster, Münster, Deutschland

**Keywords:** Vergleichstudie, E‑Bike, Verletzungsmuster, Fortschritt, Gesundheit, Helm, Comparative study, E‑bike, Injury patterns, Progress, Health, Helmet

## Abstract

**Hintergrund:**

Fahrräder sind seit vielen Jahren ein beliebtes Verkehrsmittel. Gerade in Zeiten der verstärkten Klimadiskussion ist das Fahrrad als umweltfreundliches und kostengünstiges Verkehrsmittel weiter in den Fokus gerückt. Die Radwege und Straßen werden immer voller, und neue Verkehrsmittel wie Pedelecs oder E‑Scooter kommen auf.

**Methoden:**

Es wurden insgesamt 4 Studien des Universitätsklinikum Münster zu Fahrradunfällen und die amtliche Unfallstatistik der Polizei Münster miteinander verglichen. Im Zeitraum von 2009 bis 2019 wurden 3 Studien durchgeführt, die alle Fahrradunfälle, und eine Studie, welche nur Pedelecfahrer gesondert berücksichtigt.

**Ergebnisse:**

Die Altersverteilung sowie Hauptunfallursachen sind über die Jahre hinweg nahezu gleich geblieben. Die Anzahl an Pedelecunfällen hat zugenommen. Pedelecfahrer weisen ein höheres Durchschnittsalter und einen höheren Anteil an intensivstationären Aufenthalten auf. Jedoch weist gerade diese Kohorte auch eine hohe Quote an Helmträgern auf. Insgesamt scheint die Bereitschaft zum Tragen eines Helmes gestiegen zu sein.

**Schlussfolgerung:**

Es ist zu bedenken, dass bei zunehmendem Radverkehr die Sicherheitsmaßnahmen entsprechend erhöht werden müssen. Dabei sollte sich die Unfallverhütung auf 3 große Bereiche konzentrieren: Technik, Erziehung, Durchsetzung.

## Abstract (en)

**Background:**

Bicycles have been a popular means of transport for many years. Especially in times of increased climate discussion, the bicycle has moved further into focus as an environmentally friendly and cost-effective means of transport. Bike lanes and roads are becoming more crowded and new means of transport such as pedelecs or e‑scooters are emerging.

**Methods:**

A total of four studies by Münster University Hospital on bicycle accidents and the official accident statistics of the Münster police were compared. In the period from 2009 to 2019, three studies were conducted that considered all bicycle accidents and one study that only considered pedelec riders separately.

**Results:**

The age distribution as well as main causes of accidents remained almost the same over the years. The number of pedelec accidents has increased. Pedelec riders have a higher average age and a higher proportion of intensive care stays; however, this cohort also has a high rate of helmet wearers. Overall, the willingness to wear a helmet seems to have increased.

**Conclusion:**

It should be considered that with increasing bicycle traffic, safety measures must be increased accordingly. In this respect, accident prevention should focus on three major areas, engineering, education and enforcement.

Fahrräder sind seit Jahren ein beliebtes Transportmittel. Sie bieten Vorteile für Umwelt und Gesundheit, dennoch sind Fahrradunfälle im Straßenverkehr häufig folgenschwer. Mit dem Wandel der Fahrradnutzung hin zur E‑Mobilität müssen sich die Bedingungen für Radfahrer im Sinne der Unfallverhütung weiterentwickeln. Viele Sicherheitsmaßnahmen beruhen jedoch auf den offiziellen Unfallstatistiken der Polizei, welche eine große Dunkelziffer aufweisen. In diesem Beitrag wird die Entwicklung der im Universitätsklinikum Münster (UKM) erfassten Radunfälle der vergangenen 10 Jahren betrachtet.

Im Laufe der Jahre hat sich das Radfahren zu einer Art Kultur entwickelt, und auch das Freizeitradeln nimmt immer weiter zu [5]. Diese Tendenzen wurden durch die COVID-19-Pandemie verstärkt. In einer Studie zur Fahrradnutzung in Deutschland aus Juni 2020 gaben 25 % der Teilnehmer an, ihr Fahrrad häufiger zu benutzen; 31 % gaben an, das Fahrrad als Ersatz für andere Freizeitaktivitäten zu nutzen [15].

Vor allem Pedelecs haben an Beliebtheit gewonnen. Pedelec ist die Abkürzung für „pedal electric cycle“. Als Untergruppe der E‑Bikes (alle elektrisch unterstützten Zweiräder) sind Pedelecs elektrisch unterstützte Zweiräder, die dann Unterstützung bieten, wenn der Fahrer aktiv in die Pedale tritt. *Die motorisierte Unterstützung besteht bis zu einer maximalen Geschwindigkeit von 25* *km/h und wird progressiv verringert. Eine Anfahr- bzw. Schiebehilfe bis 6* *km/h ist zulässig. Ein Mindestalter oder eine Helmpflicht für die Nutzung von Pedelecs besteht nicht *[1].

Die Zahl der E‑Bikes in deutschen Haushalten ist in den letzten 6 Jahren von 1,6 Mio. (2013) auf 5,4 Mio. (2019) gestiegen [23].

Die Vorteile des Radfahrens wie Umweltschonung und Gesundheitsförderung scheinen die Einwohner der nordrhein-westfälischen Stadt Münster schon lange überzeugt zu haben. Bereits im Jahr 2009 lag die Zahl der Fahrräder in der Stadt bei rund 400.000 und damit weit über der Einwohnerzahl (273.000) [16]. Im Jahr 2019 lebten dort über 310.000 Menschen, die mittlerweile über 500.000 Fahrräder besitzen [11]. Der Radverkehrsanteil in Münster liegt bei 39,1 %, damit ist das Fahrrad das führende Verkehrsmittel in dieser Stadt, gefolgt vom Pkw mit 29,0 % („modal split“) [3]. In dieser Arbeit wird die Stadt Münster beispielhaft für die Entwicklung von Fahrradunfällen in den letzten 10 Jahren genutzt. Sie konzentriert sich auf den Vergleich verschiedener Fahrradunfallstatistiken des UKM und der Polizei Münster von 2009 bis 2019.

## Methodik

Um die Entwicklung der Fahrradunfälle in Münster zu analysieren, wurden 4 Studien des UKM mit der offiziellen Verkehrsunfallstatistik der Polizei Münster verglichen. Alle Daten wurden mittels eines Patientenfragebogens und des Krankenhausinformationssystems erhoben. Die erste Studie wurde von Februar 2009 bis Januar 2010 in 6 Münsteraner Krankenhäusern durchgeführt und in der Zeitschrift *Injury* veröffentlicht [8]. Um alle Studienergebnisse vergleichbar zu machen, wurden hier nur die Patienten berücksichtigt, die sich in diesem Zeitraum im UKM vorstellten. Die zweite Studie „International Bicycle Accident Study (IBAS)“ startete im Mai 2012 und endete im April 2013 und wurde vom Bundesamt für Straßenverkehr veröffentlicht [4]. 23 der 25 Kliniken aus dem TraumaNetzwerk NordWest nahmen daran teil. Aus Gründen der Vergleichbarkeit wurden auch hier nur die am UKM erhobenen Daten verwendet. Die dritte Studie umfasste einen Zeitraum von November 2017 bis Mai 2019 und wurde vom UKM durchgeführt. Die Daten aus dieser Studie sind bisher nicht veröffentlicht. In einer vierten Studie wurden vom Mai 2018 bis April 2019 gezielt Patienten angesprochen, welche angaben, mit einem E‑Bike verunglückt zu sein. Es stellten sich ausschließlich Pedelecfahrer vor. Diese Studie wurde vom Bundesamt für Straßenverkehr finanziell unterstützt und veröffentlicht [24], welches daher die Rechte an den erhobenen Daten hat und uns freundlicherweise die Daten der stationären Patienten für diese Arbeit zur Verfügung stellte. Da sich die Erhebungszeiträume der 3. und 4. Studie überschneiden, wurden Patientendaten, die sowohl in der 3. als auch in der 4. Studie auftauchten, aus dem Datensatz der 3. Studie entfernt, um Doppelzählungen zu vermeiden. Dies betraf 5 Fälle.

Eine Übersicht über alle eingeschlossenen Studien, ihre Dauer und die einbezogenen Fahrradtypen ist in Tab. 1 enthalten. Die Datenanalyse und Grafikerstellung wurden mittels Excel und R‑Studio (Posit PBC, Boston, Massachusetts, USA) durchgeführt.

**Table 1:** 

Studien	Studienzeitraum	Fahrradtypen	*n*	Pedelecs, absolut	Pedelecs, prozentual (%)
Studie 1	01.02.2009–31.01.2010	Alle	452	1	0,2
Studie 2	01.05.2012–30.04.2013	Alle	329	13	4,0
Studie 3	01.11.2017–31.05.2019	Alle	108	5	4,6
Studie 4	01.05.2018–30.04.2019	Nur E‑Bikes	17	17	100,0

Alle Studien wurden von der Ethikkommission der Westfälischen Wilhelms-Universität Münster genehmigt^1^.

Um einen Vergleich zu den polizeilich erfassten Verkehrsunfällen ziehen zu können, wurden die online frei zugängliche Verkehrsunfallstatistik der Polizei in Münster aus den jeweiligen Jahren in die Auswertung einbezogen.

## Ergebnisse

Aufgrund strengerer Datenschutzbedingungen und personeller Veränderungen ist ein Rückgang der Erfassungszahlen am UKM zu verzeichnen. Dies bedeutet jedoch keinen Rückgang der Unfallzahlen in den Münsteraner Krankenhäusern; vielmehr ist davon auszugehen, dass die Unfallzahlen angesichts der steigenden Zahlen in der amtlichen Statistik ähnlich hoch oder sogar höher sein müssten (Tab. 2).

**Table 2:** 

Polizei Münster	2009	2012	2018	2019
Insgesamt	650	669	863	873
Pedelecs, absolut	–	–	74	73
Pedelecs, prozentual	–	–	8,6 %	8,4 %

**Figure 1:**
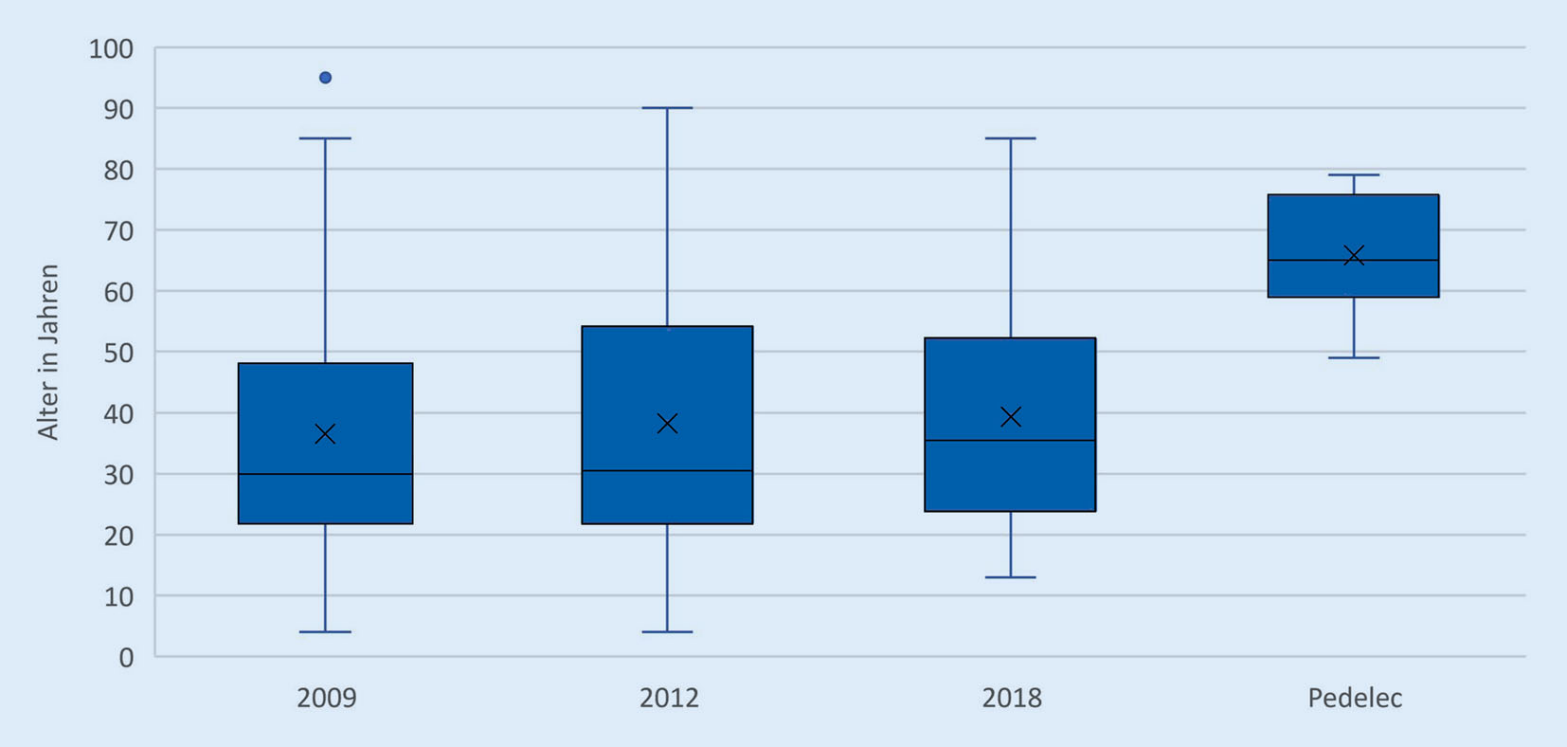

*Die Altersverteilung aller Studien ist in etwa gleich, mit einem Höhepunkt im Alter von 20 bis 29 Jahren und einem Durchschnittsalter von 35 bis 40 Jahren. Der jüngste Patient war 4 Jahre und der Älteste 95 Jahre alt. Das Durchschnittsalter der Kohorte der Pedelecfahrer ist deutlich höher mit einem Mittelwert von 66 Jahren *(Abb. 1).* Der jüngste Pedelecfahrer war 49 Jahre und der Älteste 79 Jahre alt*.

**Figure 2:**
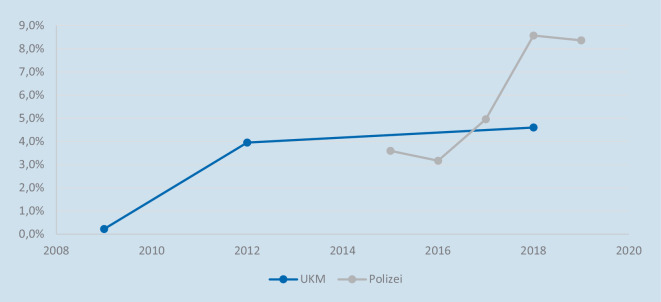

*Bei den am UKM erfassten E‑Bike-Unfällen gab es im betrachteten Zeitraum einen leichten Anstieg *(Tab. 1) *(p-Wert: <* *0,01* *%). Würde man die Fälle aus der reinen Pedelecstudie miteinberechnen, wäre dieser vermutlich deutlicher. In der amtlichen Unfallstatistik liegt der Anteil der E‑Bike-Unfälle im Jahr 2019 bei 8,36* *% und ist damit gegenüber den anfänglichen 3* *% aus dem Jahr 2015 gestiegen *(Abb. 2).

Über alle Studien hinweg ist die deutliche Hauptunfallursache der Alleinunfall, gefolgt von Kollisionen mit anderen Fahrrädern und Autos. In der offiziellen Unfallstatistik für 2019 sind die Hauptunfallursachen falsches Abbiegen, Missachtung des Abstands oder der Vorfahrt und Alkoholeinfluss. Im Jahr 2009 war die Hauptunfallursache erhöhter Alkoholeinfluss, gefolgt vom Benutzen der falschen Fahrspur und der Missachtung der Vorfahrt.

*In den klinischen Studien wurde der Alkoholeinfluss auf freiwilliger Basis abgefragt. Im Jahre 2009 und 2018 lag der Anteil der Patienten, die Alkohol konsumierten, bei rund 3* *% (2009: 3,1* *%; 2018: 2,8* *%), und im Jahre 2012 zeigte sich ein deutlicher Peak mit 16,7* *% an alkoholisiert Verunfallten (p-Wert: <* *0,01* *%). In der Pedelecstudie wurde der Alkoholkonsum nicht abgefragt. *In der amtlichen Statistik ging die Zahl der Fahrradunfälle aufgrund von Trunkenheit leicht zurück, von 69 Unfällen unter Alkoholeinfluss im Jahr 2009 auf 60 im Jahr 2019.

**Figure 3:**
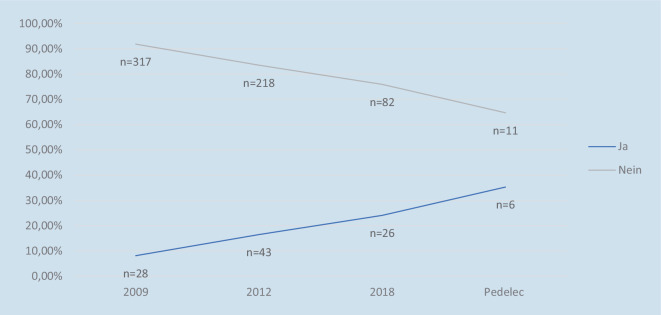

Positiv entwickelte sich die Helmnutzung in den Studien*. So lag die Quote der Helmträger im Jahre 2009 bei 8,1* *% und stieg auf 24,1* *% im Jahre 2018. Bei den Pedelecfahrern zeigt sich die höchste Rate an Helmträgern mit 35,3* *% (p-Wert:<* *0,01* *%)* (Abb. 3). Leider gibt die offizielle Unfallstatistik der Polizei Münster keine Auskunft über die Anzahl der verunglückten Helmträger.

**Figure 4:**
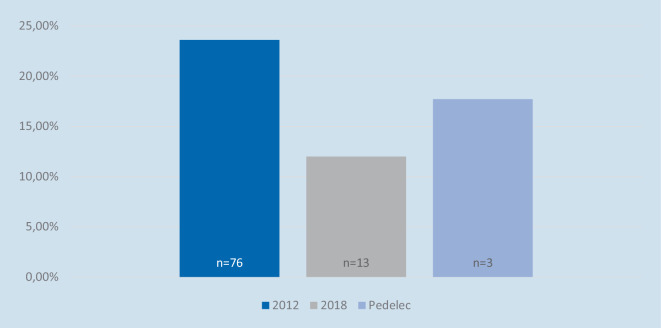

*Bei den Verletzungsmustern fällt auf, dass die Zahl der Kopfverletzungen bei den Radfahrern rückläufig ist. In der zweiten Studie aus 2012 hatten 23,6* *% der Patienten eine Kopfverletzung erlitten, im Jahre 2018 waren es nur noch 12,0* *%. Von den Pedelecfahrern erlitten 17,7* *% eine Kopfverletzung *(Abb. 4) *(p-Wert: 0,03* *%).*

*Die häufigsten Verletzungen in den 2012 und 2018 betrafen die obere Extremität *(*p*-Wert: < 0,01 %)*. Die Pedelecfahrer hatten am häufigsten eine Verletzung an der unteren Extremität *(Tab. 3) (*p*-Wert: < 0,01 %).

**Table 3:** 

Köperregion	2012 (in %)	2018 (in %)	Pedelec (in %)
Kopf	23,6	12,0	17,7
Gesicht	24,0	16,7	17,7
Hals	1,2	0,0	0,0
Thorax	8,2	3,7	5,9
Abdomen	1,5	0,9	0,0
Wirbelsäule	6,3	1,9	11,8
Obere Extremität	46,6	28,7	47,1
Untere Extremität	34,6	22,2	70,5
Äußere Verletzungen	1,2	1,9	0,0
Becken	6,4	3,7	0,0

Aus Studie 1 lagen keine vergleichbaren Daten zu Verletzungsmustern vor. Die Bewertung der Verletzungsschwere erfolgte in allen weiteren Studien über den AIS 2005.

**Figure 5:**
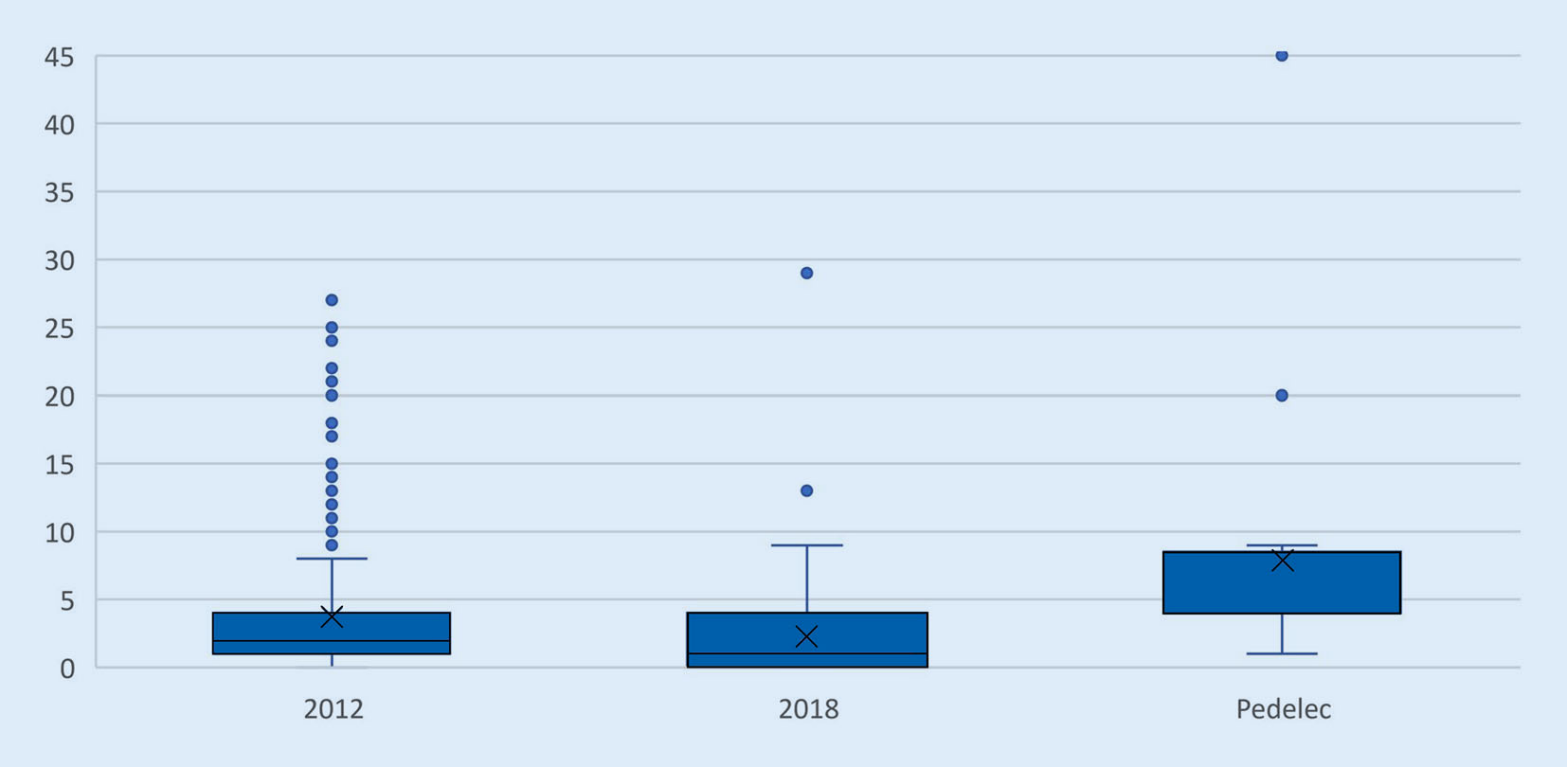

Per Definition liegt ein Polytrauma bei einem ISS (Injury Severity Score) von 16 und mehr vor. *Davon wurden 8 Patienten im Jahr 2012 und einer im Jahr 2018 erfasst. In der Kohorte der Pedelecfahrer wurden 2 polytraumatisierte Patienten aufgenommen.* Der Patient mit dem höchsten ISS von 45 gehört ebenfalls zur Kohorte der Pedelecfahrer (Abb. 5).

Laut der offiziellen Statistik der Polizei Münster liegt die Quote der schwer verletzten Radfahrer bei 17 %. Der Anteil der schwer verletzten Pedelecfahrer ist mit 38,9 % deutlich höher.

Die polizeiliche Definition eines Schwerverletzten basiert jedoch nicht auf dem ISS, sondern darauf, ob ein Patient ins Krankenhaus eingeliefert wird und 24 h verbleibt oder nicht.

**Figure 6:**
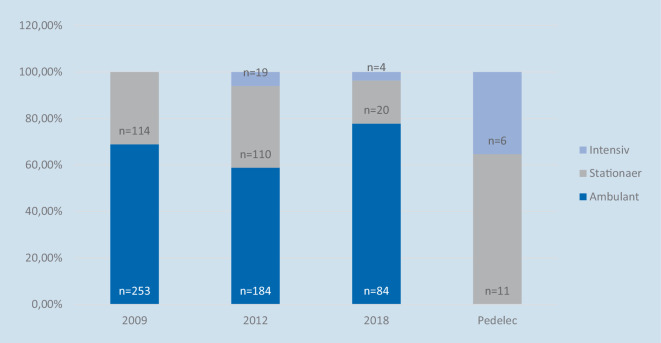

*Die Zahl der stationären Einweisungen nach einem Unfall ist in 2018 (18,5* *%) im Vergleich zu den letzten beiden Studien (2009: 30,8* *%; 2012: 35,1* *%) deutlich rückläufig *(Abb. 6) (*p*-Wert: < 0,01 %).

Wie in der Methodik beschrieben, konnten bei Pedelecfahrern nur stationäre Patienten berücksichtigt werden. Dennoch musste ein hoher Anteil der Patienten auf der Intensivstation behandelt werden. Von den 17 Patienten, die in die Studie aufgenommen wurden, mussten 6 mindestens eine Nacht auf der Intensivstation verbringen. Aus Studie 1 liegen keine Informationen über einen Aufenthalt auf der Intensivstation vor.

## Diskussion

Erst seit 2015 gibt es einen eigenen Bericht des Statistischen Bundesamtes ausschließlich für Fahrrad- und E‑Bike-Unfälle, was zeigt, wie sehr das Interesse an Fahrrädern und deren Unfallbeteiligung gewachsen ist. Die Zahl der Fahrradunfälle mit Personenschaden ist von 2009 bis 2019 um 14,68 % gestiegen. Die Gesamtzahl der Verkehrsunfälle mit Personenschaden im gleichen Zeitraum sank um 3,68 % [19]. Dies könnte durch einen starken Anstieg der Fahrradnutzung bei nichtausreichendem Verkehrsraum bedingt sein.

Die polizeiliche Verkehrsunfallstatistik 2019 der Stadt Münster zeigt, dass die Verkehrswege, insbesondere in der Innenstadt, oft stark überlastet und nicht für die derzeit über 300.000 Einwohner ausgelegt sind [12]. Besonders betroffen hiervon sind die Radfahrer, welche seit 2018 mehr als die Hälfte der Verunglückten auf Münsters Straßen ausmachen [13].

Die Polizeistatistik aus dem Jahr 2019 zeigt, dass der Abstand zwischen Kraftfahrzeugen und Radfahrern eine häufige Unfallursache bei Kollisionen ist. Es bleibt abzuwarten, ob die neue Straßenverkehrsordnung vom 28.04.2020, die beim Überholen eines einspurigen Verkehrsteilnehmers innerorts einen Mindestabstand von 1,5 m und außerorts von 2 m vorschreibt, hier präventiv wirkt [2].

Radfahren unter erhöhtem Alkoholeinfluss (> 0,16 %) ist in Deutschland eine Straftat [6]. Bundesweit ist ein Rückgang der Fahrradunfälle unter Alkoholeinfluss zu beobachten. Waren es im Jahr 2009 noch 53 Radfahrer/1000 Beteiligte, die unter Alkoholeinfluss fuhren, sank diese Zahl auf 46/1000 Beteiligte im Jahr 2019 [19]. Zu bedenken sind hier die hohe Dunkelziffer und die Tatsache, dass es sich um ein Kontrolldelikt handelt. Die Studie des Bundesamts für Straßenverkehr zeigte, dass Radfahrer, die unter Alkoholeinfluss standen, eine höhere Wahrscheinlichkeit hatten, ins Krankenhaus eingeliefert oder intensivmedizinisch versorgt zu werden [4].

Alleinunfälle tauchen in den von der Polizei erfassten Daten nur sehr selten auf, da die Polizei oft nicht zu Radunfällen gerufen wird, an denen keine anderen Personen beteiligt sind, und der Sachschaden an Fahrrädern zumeist weniger schwerwiegend ist als an Autos. Im Jahr 2009 zeigten Juhra et al. eine deutliche Diskrepanz zwischen der Zahl der von der Polizei erfassten Fahrradunfälle und der Zahl der verunglückten Radfahrer, die sich im Krankenhaus vorstellten. 67,9 % der Fahrradunfälle von Patienten, die sich selbst im Krankenhaus vorstellten, wurden von der Polizei nicht erfasst [8]. Die daraus resultierende hohe Dunkelziffer von Fahrradunfällen in vielen Statistiken, die auf den Daten polizeilich erfasster Unfälle beruhen, wie z. B. die Statistiken des Bundesamtes für Verkehr, ist problematisch, da diese Statistiken oft die Grundlage für politische und infrastrukturelle Entscheidungen sind.

Die Zahl der verunglückten Pedelecfahrer ist in den letzten Jahren angestiegen. Bundesweit lag der Anteil der verunglückten Pedelecfahrer im Jahr 2015 bei 3,76 % und hat sich in nur 4 Jahren auf 12,63 % (2019) erhöht [17, 18]. Dies könnte u. a. auf die zunehmende Beliebtheit von Pedelecs in der Bevölkerung zurückzuführen sein. Im Jahr 2009 wurden ca. 150.000 Elektrofahrräder/Jahr verkauft. Im Jahr 2019 waren es bereits 1,36 Mio. Elektrofahrräder, und dieser Trend hält an [20]. Im Jahre 2020 wurden 43,4 % mehr E‑Bikes verkauft als noch 2019 [22]. Da hiermit der Anteil von Elektrofahrrädern im Straßenverkehr weitersteigt, liegt ein Anstieg der Unfallzahlen nahe.

Weiss et al. kamen 2013 zu dem Ergebnis, dass das Pedelec keinen Einfluss auf das Unfallrisiko und die Schwere der Verletzungen hat. Hier zeigte sich eine Tendenz, dass Pedelecfahrer über 65 Jahre häufiger ins Krankenhaus eingeliefert wurden als solche unter 65 Jahren. Bei den stationären Aufnahmen gab es jedoch keinen Unterschied zwischen den über 65-Jährigen, die ein herkömmliches Fahrrad fuhren, und den Pedelecfahrern. Es wurde daher vermutet, dass das höhere Durchschnittsalter und die erhöhte Anzahl an Komorbiditäten für die stationären Behandlungen verantwortlich sind [21]. Lefarth et al. zeigten in ihrer Studie aus dem Jahr 2021, dass Pedelecfahrer signifikant mehr stationäre Aufnahmen (Fahrrad: 34 %; Pedelec: 53 %) und Intensivbehandlungen (Fahrrad: 1 %; Pedelec 7 %) sowie einen signifikant höheren ISS-Score (Fahrrad: 3,4 Punkte; Pedelec: 5,2 Punkte) in dieser Kohorte aufwiesen. Allerdings gab es auch hier bei den Pedelecfahrern ein höheres Durchschnittsalter und mehr Komorbiditäten, was für die stationären Aufnahmen und schwereren Verletzungen ausschlaggebend sein könnte [10]. Auch die Ergebnisse der vorliegenden Studien scheinen intensivmedizinische Behandlungen in der Kohorte der Pedelecfahrer häufiger notwendig zu sein als in den anderen Kohorten. Allerdings ist zu beachten, dass die Zahl der Studienteilnehmer in der Pedelecstudie kleiner ist als in den anderen Studien und somit die Repräsentativität fraglich ist.

*Eine forsa-Umfrage aus 2013 zeigt, dass v. a. Senioren (zwei Drittel der Befragten) einen Ratschlag ihres Arztes bezüglich der Fahreignung annehmen würden, jedoch wurden in der Umfrage gerade mal 4* *% von ihrem Hausarzt auf die Fahrtauglichkeit angesprochen *[14]*. Dies gilt nicht nur für Rad- und Pedelecfahrer, sondern auch für Pkw-Fahrer.*

Der leichte Anstieg der Helmnutzung bei den verunglückten Radfahrern im Jahr 2018 lässt hoffen, dass die generelle Bereitschaft zum Tragen eines Helms bei den Münsteraner Radfahrern gestiegen ist. Möglicherweise haben die verstärkten Aufklärungsprogramme der Polizei und der Stadt Münster in den vergangenen Jahren dazu beigetragen, dass die Helmnutzung in den Fokus gerückt und die Akzeptanz des Helmtragens in der Münsteraner Bevölkerung gestiegen ist [9]. Erfreulich ist v. a., dass die Kohorte der Pedelecfahrer eine sehr hohe Helmtragequote aufweist, da gerade sie im Falle eines Sturzes ein erhöhtes Risiko für Hirnblutungen tragen [7].

## Limitationen

Um die Aussagekraft der Untersuchungsergebnisse zu erhöhen, wären u. a. folgende Änderungen notwendig:

- Betrachtung anderer Städte mit geringerem Fahrradanteil im Straßenverkehr,
- Vergrößerung der Stichprobe v. a. hinsichtlich der Pedelecnutzer,
- objektive Angaben zum Alkoholkonsum,
- *Anpassung der Gruppenstärke bei hier vorliegenden großen Unterschieden in der Gruppengröße (Pedelec n* *=* *17; 2009 n* *=* *452).*

## Fazit für die Praxis

- Eine effektivere Vernetzung zwischen den Krankenhäusern und der Polizei führt zu einer Reduzierung der Dunkelziffer, lässt Ursachen und Zielgruppen genauer bestimmen und führt so zu einer Effektivitätssteigerung präventiver, aber auch repressiver Verkehrssicherheitsmaßnahmen.
- Zur Reduzierung der Dunkelziffer und zur Objektivierung bei Fahrradverunfallten hinsichtlich des Alkoholkonsums wäre in zukünftigen Studien eine aktivere Ansprache der Thematik bei Krankenhausbehandlung und Testung mittels Blutanalyse wünschenswert, um aussagekräftigere Daten über Verletzungsschwere und -muster bei alkoholisierten Radfahrern zu erhalten.
- Die Unfallverhütung bedarf einer ganzheitlichen, kooperativen Verkehrssicherheitsstrategie. Hierzu zählen die Analyse (Unfallforschung/-untersuchung), die Verkehrsraumgestaltung („engineering“), die Prävention („education“), die Verkehrsüberwachung („enforcement“) und eine zielgruppenadäquate Sicherheitskommunikation.
- Eine Beratung der Patienten könnte durch den Hausarzt hinsichtlich der *Fahrtauglichkeit *aus medizinischer Sicht erfolgen, z. B. durch aktive Bewerbung der verschiedenen polizeilichen Verkehrssicherheitsübungen und anderen Partner (z. B. ADFC) zu den Besonderheiten von elektrisch unterstützten Fahrrädern, ggf. in den Pedelecverkaufsstellen.

## Footnotes

1 Ethikantragsnummern: Studie 1: Die Ethiksantragsnummer ist nach 10-jähriger Archivierung verfallen. Studie 2: 2012-171-f‑S Studie 3: Az.: 2017-281-b‑S; Studie 4: Az.: 2018-198-f‑S.

## References

[1] Allgemeiner Deutscher Fahrrad-Club EV Rechtliche Rahmenbedingungen. https://www.adfc.de/artikel/rechtliche-rahmenbedingungen. Zugegriffen: 8. Sept. 2022

[2] Allgemeiner Deutscher Fahrrad-Club Nordrhein-Westfalen-Kreisverband Bottrop E. V. Was ist beim Überholen von Radfahrern zu beachten? https://www.adfc-nrw.de/kreisverbaende/kv-bottrop/radverkehr/verkehrsregeln/ueberholen-von-radfahrern.html. Zugegriffen: 8. Mai 2021

[3] Amt für Stadtentwicklung Stadtplanung Verkehrsplanung Abteilung Verkehrsplanung (2016). 3. Nahverkehrsplan Stadt Münster.

[4] Below A (2016). Verkehrssicherheit von Radfahrern – Analyse sicherheitsrelevanter Motive, Einstellungen und Verhaltensweisen. Mensch Sicherh.

[5] Bracher T, Hertel M (2014). Radverkehr in Deutschland Zahlen, Daten, Fakten.

[6] Bundesbussgeldkatalog (2021). Promillegrenze auf dem Fahrrad.

[7] Gioffre-Florio M, Murabito LM, Visalli C (2018). Trauma in elderly patients: a study of prevalence, comorbidities and gender differences. G Chir.

[8] Juhra C, Wieskotter B, Chu K (2012). Bicycle accidents—do we only see the tip of the iceberg? A prospective multi-centre study in a large German city combining medical and police data. Injury.

[9] Kersten R, Hartz PDIB (2017). Wirkungskontrolle der Maßnahmen zur Verbesserung der Verkehrssicherheit in Münster.

[10] Lefarth TL, Poos H, Juhra C (2021). Pedelec users get more severely injured compared to conventional cyclists. Unfallchirurg.

[11] Marketing Stadt Münster Mit dem Fahrrad durch Münster. https://www.stadt-muenster.de/tourismus/fahrradstadt. Zugegriffen: 8. Mai 2021

[12] Münster Polizei (2020). Verkehrsunfallstatistik 2019.

[13] Münster Polizeipräsidium (2019). Verkehrsunfallstatistik 2018 – Stadt Münster.

[14] Schoch S, Kenntner-Mabiala R (2021). Verkehrssicherheitsberatung älterer Kraftfahrerinnen und -fahrer in der hausärztlichen Praxis Bestandsaufnahme. Mensch Sicherh.

[15] Sinus Markt- Und Sozialforschung (2021). Fahrrad-Monitor Deutschland Corona-Befragung 2020.

[16] Stadt Münster Stadtplanungsamt Presse- Und Informationsamt (2009). Fahrradhauptstadt Münster Alle fahren Rad: gestern, heute, morgen.

[17] Statistisches Bundesamt (2016). Kraftrad- und Fahrradunfälle im Straßenverkehr 2015.

[18] Statistisches Bundesamt (2020). Verkehrsunfälle Kraftrad- und Fahrradunfälle im Straßenverkehr 2019.

[19] Statistisches Bundesamt (2021). Verkehrsunfälle Zeitreihen 2020.

[20] Wachotsch U, Kolodziej A, Specht B (2014). E-Rad macht mobil Potenziale von Pedelecs und deren Umweltwirkung.

[21] Weiss R, Juhra C, Wieskotter B (2018). How probable is it that seniors using an E-bike will have an accident?—A new health care topic, also for consulting doctors. Z Orthop Unfall.

[22] Zweirad-Industrie-Verband (2021). Zahlen – Daten – Fakten zum deutschen Fahrrad- und E-Bike Markt 2020 Fahrradindustrie mit Rückenwind – Großes Wachstum bei Absatz und Umsatz.

[23] Zweirad-Industrie-Verband (2020). Zahlen – Daten – Fakten zum Fahrradmarkt in Deutschland 2019.

[24] Platho C, Horn H-P (2021). Analyse der Merkmale und des Unfallgeschehens von Pedelecfahrern. Berichte der Bundesanstalt für Straßenwesen.

